# The Use of Inhibitors of Tyrosine Kinase in Paediatric Haemato-Oncology—When and Why?

**DOI:** 10.3390/ijms222112089

**Published:** 2021-11-08

**Authors:** Agnieszka Kaczmarska, Patrycja Śliwa, Monika Lejman, Joanna Zawitkowska

**Affiliations:** 1 Student Scientific Society, Laboratory of Genetic Diagnostics, Medical University of Lublin, Gębali 6, 20-093 Lublin, Poland; agnieszkakacz70@gmail.com (A.K.); sliwa.pat@gmail.com (P.Ś.); 2 Laboratory of Genetic Diagnostics, Medical University of Lublin, Gębali 6, 20-093 Lublin, Poland; 3 Department of Pediatric Hematology, Oncology and Transplantology, Medical University of Lublin, Gębali 6, 20-093 Lublin, Poland

**Keywords:** tyrosine kinase inhibitors, TKIs, paediatric oncology

## Abstract

The fundamental pathophysiology of malignancies is dysregulation of the signalling pathways. Protein tyrosine kinases (PTKs) are among the enzymes which, if mutated, play a critical role in carcinogenesis. The best-studied rearrangement, which enhances PTK activity and causes atypical proliferation, is *BCR-ABL1*. Abnormal expression of PTKs has proven to play a significant role in the development of various malignancies, such as chronic myelogenous leukaemia, brain tumours, neuroblastoma, and gastrointestinal stromal tumours. The use of tyrosine kinase inhibitors (TKIs) is an outstanding example of successful target therapy. TKIs have been effectively applied in the adult oncology setting, but there is a need to establish TKIs’ importance in paediatric patients. Many years of research have allowed a significant improvement in the outcome of childhood cancers. However, there are still groups of patients who have a poor prognosis, where the intensification of chemotherapy could even cause death. TKIs are designed to target specific PTKs, which lead to the limitation of severe adverse effects and increase overall survival. These advances will hopefully allow new therapeutic approaches in paediatric haemato-oncology to emerge. In this review, we present an analysis of the current data on tyrosine kinase inhibitors in childhood cancers.

## 1. Introduction

**Figure 1:**
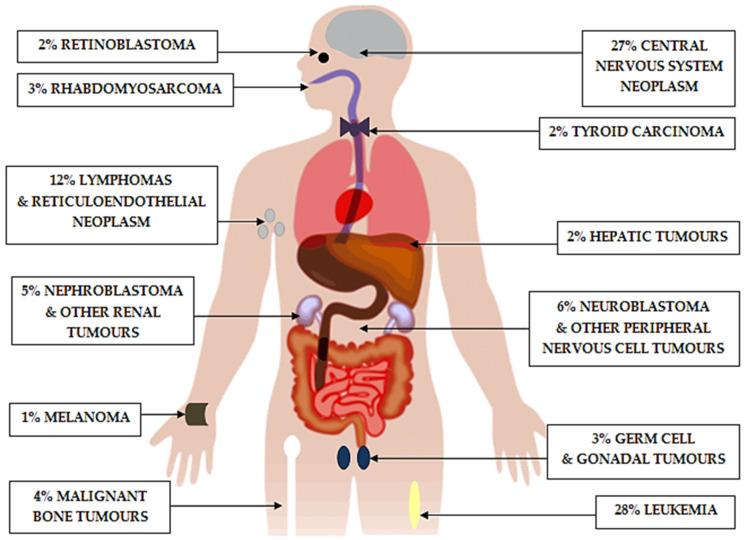
Cancer case distribution in the United States (2013–2017) in children from birth to 14 years old, according to the American Cancer Society.

In recent years, we have observed tremendous progress in paediatric haemato-oncology [1]. Malignant neoplasms are the third leading cause of death (after injury-related causes and motor vehicle crashes) in children, and they represented 9% of overall deaths in the United States of America (USA) in 2016 [2]. According to the American Cancer Society report, it is predicted that approximately 10,500 children in the USA under the age of 15 will be diagnosed and 1190 will die from cancer in 2021. The World Health Organisation (WHO) estimates that each year, 400,000 children and adolescents are diagnosed with cancer. The 5-year survival for all children aged 5–14 in the period 1975–1978 was 59%, and it increased to 80.6% in 1999–2002 [3]. In high-income countries, where there is easy access to medical care, more than 90% of children with cancer recover. However, in low- and middle-income countries, the results are significantly worse and only around 15–45% of children are cured [4]. The most common cancer in children under 15 years old is leukaemia (28%), followed by brain tumours (27%), and, thirdly, lymphoma (9%) (Figure 1) [5]. Despite intensive therapy, the cure rate of childhood cancers is still unsatisfactory. A strategy by which it will be possible to achieve higher survival rates among children with cancer requires, above all, new treatment options. In view of the small target group and the ethical complexities of drug testing in children, paediatric patients are often forgotten in clinical trials [6]. The development of medicine has produced integrated multidisciplinary care for paediatric oncology patients, and the concept of tailoring treatment to an individual patient is quickly becoming a reality. These include target therapies, which are drugs that block the growth and spread of cancer by interfering with specific molecules [7]. Representatives of target therapies are tyrosine kinase inhibitors. In this review, we will outline the use of tyrosine kinase inhibitors and highlight areas for further research in childhood haemato-oncology.

## 2. Historical Perspective and Current Treatment Challenges

The first ever chemotherapy drug that proved to be effective was aminopterin, used by Sidney Farber in 1948 to treat acute lymphoblastic leukaemia (ALL) [8,9]. Seven years later, the same doctor and his colleagues achieved the first remission of Wilms’ kidney tumour in a paediatric patient. In addition to surgery and radiotherapy, they used the antibiotic actinomycin D [10]. Since then, paediatric oncologists have joined surgeons and radiation oncologists to form a cooperative group; together, they are trying to develop new therapeutic approaches [11]. Anticancer drugs include chemotherapy (classic drugs that are toxic to cells), hormone therapy (drugs that act by inhibiting or enhancing the action of certain hormones), target therapy (drugs that are molecularly targeted), and finally, immunotherapy (drugs that stimulate the immune system). Chemotherapy is a strong pillar in the treatment of paediatric malignancies; however, some drugs are still used off-label, which means that there are no indications for their use accepted by regulatory bodies [12,13,14]. Cytotoxic drugs inhibit the division of the rapidly growing cancerous cells, but they also affect normal cells, which cause characteristic adverse events [15]. Chemotherapy is administered according to a multidrug protocol, which is divided into separate phases (induction, consolidation, and maintenance) and takes usually 2 to 3 years to complete. Since children differ radically from adults in the way they absorb and metabolise drugs, toxicity is a major problem in childhood chemotherapy. An additional disadvantage of this therapy is the frequent phenomenon of drug resistance [16]. As mentioned above, chemotherapy is associated with side effects, which often induce the failure of oncology treatment, which supports the need to find new, effective solutions [17]. Advances in molecular science and an understanding of genetic predispositions to tumour formation have produced a new treatment option called target therapy, which influences the activity of specific enzymes that are involved in carcinogenesis. This approach not only reduces the toxicity of the therapy but is also more specific to tumour cells and has already led to beneficial clinical effects [18].

## 3. Protein Tyrosine Kinase

**Figure 2:**
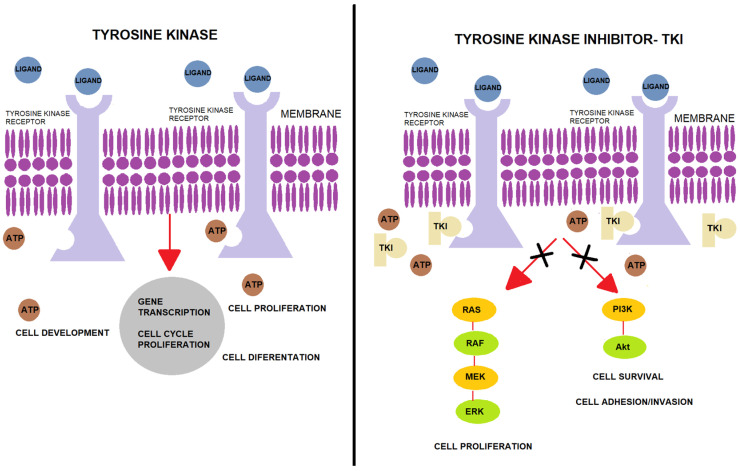
Mechanism of action of tyrosine kinase and tyrosine kinase inhibitors. Normally, tyrosine kinase transfers a phosphate group from ATP to specific intracellular proteins, which is necessary for gene transcription and cell proliferation. If a TKI is attached instead of ATP, there is a disturbance in the process, which can lead to apoptosis. TKI, tyrosine kinase inhibitor; Akt, serine/threonine kinase 1; ATP, adenosine triphosphate; ERK, extracellular signal-regulated kinases; MAPK, mitogen-activated protein kinase; PI3K, phosphoinositide 3-kinase; RAF, proto-oncogene; RAS, rat sarcoma virus protein family.

Protein tyrosine kinases (PTKs) are a class of proteins with tyrosine kinase activity and are among the most important signalling enzymes in the process of cell signal transduction [19]. PTKs catalyse the transfer of adenosine triphosphate (ATP) to the tyrosine residues of the substrate protein. ATP mediates the transfer of energy by phosphorylation, which results in further activation of signal transduction into the cytoplasm of cells. Phosphorylation of proteins by PTK is also an important mechanism of cellular proliferation, differentiation, development, apoptosis, and death. Deregulation or overexpression of PTK has been shown to play a role in carcinogenesis, and PTK has become a target for drug development and a hot spot for antitumour medicine research. Rapid progress in molecular research has gradually clarified the processes concerning cancer cells and has helped to establish a target point for treatment. PTKs can be separated into two categories: receptor PTKs (RTKs) and non-receptor PTKs (NRTKs). Analysis of the human genome has shown that there are 518 kinase genes in the human body, among which, 90 PTKs have been identified, including 58 types of RTK and 32 types of NRTK [20,21,22]. RTKs’ function is the transduction of extracellular signals into the cell, while NRTKs are responsible for intracellular communication. RTK-mediated signals are crucial in the regulation of various cellular processes such as metabolism, cell growth, differentiation, and migration. Neoplastic cells take over the function of RTK signalling, which results in a disturbance of the abovementioned developmental functions. RTKs are similar to each other in their molecular structure, which consists of three elements: (1) an extracellular domain that can bind specific ligands, (2) a single transmembrane helix, and (3) an intracellular region that contains a protein tyrosine kinase domain. Ligand binding induces dimerization of these receptor tyrosine kinases, which cause autophosphorylation of their cytoplasmic domains and activate PTK. However, NRTKs differ from RTKs, as they lack receptor-like features such as an extracellular ligand-binding domain and a transmembrane helix. NRTKs play a broad range of roles in cell signalling, including the regulation of gene expression, inhibition of cell growth via the stimulation of nuclear TKs (such as ABL), and regulation of cell adhesion and proliferation. Based on knowledge of PTK’s mechanism of action, tyrosine kinase inhibitors (TKIs) were developed. The mechanism of TKI is competitive inhibition of ATP at the catalytic tyrosine kinase binding site [23,24,25]. By competing for the ATP binding site of tyrosine kinase, TKIs reduce PTK phosphorylation, which, in effect, stops cancer cell proliferation (Figure 2). Moreover, TKIs can inhibit the repair of tumour cells and block cell division in the G1 phase, which induces apoptosis. Based on their main targets, TKIs can be divided into epidermal growth factor receptor (EGFR) inhibitors, vascular endothelial growth factor (VEGFR) inhibitors, anaplastic lymphoma kinase (ALK) inhibitors, and *BCR-ABL1* inhibitors [26]. Paediatric solid tumours, such as osteosarcoma, gastrointestinal stromal tumours (GIST), synovial sarcoma, and neuroblastoma, have an overexpression of the platelet-derived growth factor receptor (PDGFR), while Ewing sarcoma and neuroblastoma have an overexpression of c-Kit. Based on these facts, it was concluded that the inhibition of PTKs may affect antitumour activity.

## 4. Tyrosine Kinase Inhibitors

Although TKIs share the same method of action, they differ from each other in the spectrum of their targeted kinases, their pharmacokinetics, and their adverse effects [27]. Since the approval of the first TKI, imatinib, for the treatment of chronic myeloid leukaemia, several potent and well-tolerated TKIs targeting EGFR, ALK, ROS1, HER2, MET, MEK, NTRK, VEGFR, RET FGFR, PDGFR, and KIT have emerged. Moreover, some TKIs are multi-targeted, which increases the chance of a cure, but, on the other hand, also increases the toxicity. TKIs can also be useful in combination with traditional chemotherapy. Many generations of TKIs have been created so far, and each of them can be selected for specific malignancies. TKIs can also be classified as Type I or Type II inhibitors, according to whether they recognise an active or inactive kinase conformation. Dasatinib is classified as a Type I inhibitor, imatinib, nilotinib, and ponatinib belong to Type II, and bosutinib has features of both types. Type I inhibitors have less selectivity for binding and directly compete for binding with ATP. Type II inhibitors are ATP-competitive, have stricter binding requirements, and are more likely to have mutations [28]. The choice of TKIs in therapy is most often based on international treatment protocols or clinical and empirical practice. The problem occurs at the moment of resistance to a given TKI. In this case, the most logical option seems to be the use of TKIs which the patient has had no contact with and thus could not have developed resistance to. Redaelli et al. investigated the activity of imatinib, dasatinib, bosutinib, and nilotinib against a panel of 18 mutated forms of *BCR/ABL1* associated with imatinib resistance in CML and ALL. The relative resistance (RR) values were assessed using four categories: sensitive (RR ≤ 2), moderately resistant (RR 2.01 to 4), resistant (RR 4.01 to 10), and highly resistant (RR > 10). Among the 18 mutations tested, only one (*G398R*) showed an RR below 1, and this was found against imatinib. The results of the remaining TKIs were more heterogeneous: RR values greater than 2 were observed in 8 of 18 bosutinib mutants, 10 of 18 for dasatinib, and 13 of 18 for nilotinib and imatinib. Imatinib, bosutinib, and nilotinib, in addition to T315I, showed one highly resistant mutant (*E255V* for imatinib and nilotinib, *V299L* for bosutinib), while dasatinib showed a high RR only against *T315I* [29].

### 4.1. First-Generation TKIs: Imatinib

In 2001, the FDA (Food and Drug Administration) approved the first TKI, imatinib (STI571), which revolutionised cancer therapy. Since then, intensive research to obtain the most effective and well-tolerated drugs has been conducted [30]. Imatinib is an oral signal transduction inhibitor with high selectivity that targets several PTKs, especially all the ABL tyrosine kinases, including *BCR-ABL1*, platelet-derived growth factor receptor (PDGFR), and the stem cell factor receptor (c-KIT). Imatinib is metabolised in the liver, mainly by the cytochrome P450 CYP3A4 isoform, which is involved in degradation to form the main active metabolite, *N*-desmethyl imatinib. Imatinib has been shown to have remarkable clinical activity in patients with chronic myeloid leukaemia (CML), acute lymphoblastic leukaemia (ALL), and GIST. Treatment with imatinib is well-tolerated and has a low incidence of severe side effects. The most common adverse reactions are mild to moderate oedema, diarrhoea, nausea, skin rashes, and myelosuppression. Additionally, in some cases, imatinib can cause hypophosphataemia. Due to this fact, routine measurement of phosphate and vitamin D levels during imatinib therapy is recommended. Imatinib resistance is becoming a growing concern. It can occur through various mechanisms such as *BCR/ABL1* amplification and the low bioavailability of imatinib. However, in most patients, the reduced efficacy of imatinib therapy is caused by point mutations in the protein sequence. Currently, there are over 50 known mutation sites and over 70 single mutations that are responsible for imatinib resistance in CML patients. There are four regions of mutation: the phosphate binding loop (P loop), the catalytic domain, the imatinib binding site, and the activation loop. Mutations may affect drug–protein interactions either directly or indirectly if their presence shifts the thermodynamic equilibrium from an inactive to an active conformation of the enzyme [29]. Nevertheless, in these cases of resistance, second- or third-generation TKI therapy is more effective.

### 4.2. Second-Generation TKIs: Dasatinib

Dasatinib is an oral, short-acting, small-molecule inhibitor of multiple PTKs. Initially, it was designed to inhibit ABL and Src, but it also shows activity towards other kinases, including c-KIT, PDGFR-α, PDGFR-β, and ephrin receptor kinases. The inhibition of migration, invasion, and cell adhesion by dasatinib has frequently been reported, but some preclinical studies have suggested that dasatinib induces apoptosis in only a small subset of cell lines. Dasatinib is a very effective treatment for *BCR-ABL1*-driven diseases such as CML and Philadelphia-chromosome-positive acute lymphoblastic leukaemia (Ph+ ALL). Dasatinib is a dual specific inhibitor of SRC and ABL that is structurally unrelated to imatinib and is capable of binding and inhibiting both the active and inactive ABL conformation, resulting in 100–300 times greater activity than imatinib. Since dasatinib is not only a *BCL/ABL1* kinase inhibitor but also a Src family tyrosine kinase inhibitor, dasatinib may impose less stringent conformational requirements on ABL for kinase inhibition, and it has been characterised as having greater potency than imatinib. Recently, it was approved by the FDA for the treatment of children in the chronic phase of CML. However, recently, a mechanism of dasatinib resistance has been identified [29].

### 4.3. Third-Generation TKIs: Ponatinib

Ponatinib is a third-generation TKI and has high effectiveness against the mutated forms of BCR-ABL1, including T315, which is responsible for resistance to the first- and second-generation TKIs [31]. Ponatinib also inhibits the FGFR, FLT3, and Src family kinases. The metabolism of ponatinib involves CYP3A4 and, to a lesser extent, CYP2C8, CYP2D6, and CYP3A5. Based on clinical trials, ponatinib was approved for the second-line treatment of CML and Ph+ ALL. Ponatinib was temporarily suspended in 2013 because of the occurrence of thrombotic cardiovascular events. Since then, different investigators have analysed the baseline characteristics of patient candidates for ponatinib therapy. Recent studies have shown that ponatinib induces some rare but possible side effects, such as platelet aggregation, cutaneous toxicity, stroke, and/or heart attack [32]. The largest group of third-generation TKIs is the generation of epidermal growth factor receptor (EGFR) inhibitors: osimertinib, olmutinib, nazartinib, avitinib, and naquotinib. However, due to the fact that they are used as therapy for the treatment of non-small-cell lung carcinoma, which is rare in children, there are no studies involving paediatric patients (Table 1).

## 5. The Use of TKIs in Paediatric Haemato-Oncology

### 5.1. CML

The best example of the outstanding effectiveness of TKIs is their use in the treatment of CML. The pathophysiological background of CML is related to the Philadelphia chromosome, which is a result of translocation of the *BCR* gene from chromosome 22 with the *ABL1* gene on chromosome 9. The fusion product, the *BCR-ABL1* gene, is a tyrosine kinase, which disturbs the downstream signalling pathways, causing enhanced proliferation, differentiation arrest, and resistance to cell death. Allogeneic haematopoietic stem cell transplantation (HSCT) used to be the first-line curative treatment for CML until imatinib pointed oncology in a new direction. Imatinib mesylate binds the inactive moiety of the *BCR-ABL* kinase and completely blocks its ATP binding site. This leads to inhibition of the tyrosine phosphorylation of proteins that are involved in the intracellular signal transduction that *BCR-ABL1* mediates [73]. The introduction of imatinib changed the prognosis of CML tremendously [74]. This remarkable success was the result of the Phase III International Randomised Study of Interferon and STI571 (IRIS) in newly diagnosed chronic-phase CML, where the efficacy of imatinib was compared with interferon alpha combined with low-dose cytarabine. The data showed that imatinib at a dose of 400 mg once a day was more active and was associated with fewer side effects than interferon alpha plus cytarabine. After 18 months of follow-up, the estimated rate of complete cytogenetic response was 76.2% in the imatinib group compared with 14.5% in the group that received interferon alpha plus cytarabine [75]. Before the research of Suttorp et al., studies had been performed on small groups of patients with limited clinical benefits [34,76]. However, the results from a Phase III trial suggested that imatinib could be a front-line treatment in children and adolescents with CML. A total of 156 patients (age range: 1.3–18.0 years) with newly diagnosed CML (n = 146, chronic phase; n = 3, accelerated phase; n = 7, blastic phase (CML-BP)) received imatinib upfront (300, 400, and 500 mg/m^2^, respectively). In 2006, European Leukaemia Net (ELN) developed the definitions of complete haematological response (CHR), complete cytogenetic response (CCyR), and major molecular response (MMR), which standardised the use of terms, allowing researchers to easily compare the results of different studies. In the study mentioned above, CHR by Month 3 was achieved in 98% of patients, CCyR by Month 12 in 63%, and MMR by Month 18 in 59% of the patients. A total of 28 patients (19%) underwent HSCT. This clinical trial confirmed the observation from previous smaller studies that imatinib is highly effective in children and adolescents with CML. The second-generation TKIs are increasingly gaining importance. Dasatinib, nilotinib, and bosutinib have shown higher response rates and fewer adverse events compared with imatinib. Nilotinib has been used for over 10 years in CML therapy. The drug not only shows superior effectiveness in both first- and second-line CML therapy, but also leads to deep and long-lasting remission [77].

### 5.2. Ph+ ALL and Ph-Like ALL

Acute lymphoblastic leukaemia is the most common cancer in childhood and represents roughly up to 80% of all cases in children [78]. The outcome of children with ALL has strongly increased over the last few decades, and now the survival rate is over 90% in developed countries [79]. There are many subtypes of ALL, one of which is Ph+ ALL, which is present in 3–4% of ALL cases. Ph+ ALL is defined by the t(9;22)(q34;q11) translocation that produces *BCR-ABL1*, a constitutively active tyrosine kinase. Haematopoietic stem cell transplantation (HSCT) is the gold standard therapy in Ph+ ALL patients, but recently, TKIs have revolutionised the treatment approach [80]. The results of the study by the Children’s Oncology Group (COG) and the European Study of Postinduction Treatment of Ph-Positive ALL (EsPhALL) consortium suggested that the combination of chemotherapy and TKIs, without HSCT in the first remission (CR1), could be an effective treatment approach for Ph+ ALL paediatric patients. Three trials (EsPhALL2004, EsphALL2010, and EsphALL2017) have been conducted [81,82]. In EsphALL2004, patients were divided into three groups. Patients who were allocated to the good-risk group were randomly assigned to receive imatinib (300 mg/m^2^ per day) plus chemotherapy, or chemotherapy alone. The poor-risk group received post-induction imatinib plus chemotherapy. In the good-risk group treated with imatinib and in the good-risk group treated without imatinib, overall survival (OS) was 78.5%, compared with 62.9% in the poor-risk group. Due to the satisfactory results of EsphALL2004, further research was undertaken. In EsphALL2010, imatinib was started on the 15th day of the induction phase (in EsphALL2004, imatinib was administered only after induction) and the drug administration was prolonged until the end of therapy. The good-risk group achieved higher OS (75.7%) compared with the poor-risk group (63.6%). However, as a result of the long exposure to imatinib, side effects and toxicity turned out to be a major concern. The EsPhALL2017 study is currently ongoing and aims to balance treatment-related toxicity without affecting high rates of favourable outcomes [83].

Based on gene expression profiling studies, a new subtype of ALL has been described, which is named Philadelphia chromosome-like acute lymphoblastic leukaemia (Ph-like ALL), and which accounts for around 15% of B-ALL cases [84]. Ph-like ALL’s gene expression profile is extremely similar to that of Ph+ ALL but lacks the exact *BCR-ABL1* fusion [85]. Ph-like ALL harbours a diverse range of genetic alterations, such as activation of cytokine receptor genes and kinase signalling pathways, which makes it possible to treat it with TKI therapy. The role of TKI in Ph-like ALL patients has not been established yet, but promising results in Ph+ ALL patients have suggested that this approach could be a therapeutic success. The results of the study conducted by Tanasi et al. indicated that patients with ABL-class kinase fusion treated with TKI as first-line therapy or at relapse revealed better minimal residual disease (MRD) responses and a higher OS rate [86]. To sum up, several questions remain to be addressed, including the choice of TKI and time of treatment.

## 6. TKIs in Solid Tumours

The Children’s Oncology Study Group performed a Phase II study of imatinib in children and young adults with solid tumours, such as recurrent or refractory osteosarcoma, Ewing sarcoma, synovial sarcoma, desmoplastic small round cell tumour, neuroblastoma, or GIST. A total of 70 patients with the abovementioned diseases who enrolled in the study received imatinib at a dose of 440 mg every day during the treatment. Only one patient with Ewing sarcoma, who received a total of six courses of imatinib, had a partial response after two courses. This study’s results suggested that imatinib is not sufficient for solid tumour therapy [87].

### 6.1. GIST

Gastrointestinal stromal tumours are mesenchymal tumours of the gastrointestinal tract. GIST expresses the cell-surface transmembrane receptor KIT, which has tyrosine kinase activity and is the protein product of the *KIT* proto-oncogene [88,89]. However, GIST typically occurs in adults over the age of 40 years and is very rare in children. One of the main biological differences between adults and children is that paediatric patients lack activating mutations in the oncogenes KIT or platelet-derived growth factor receptor alpha (PDGFRA), which drive tumour formation in adults [90]. Overall, the GIST incidence rate is 6.5–14.5/million per year and 0.02 per million children under the age of 14 years, according to the report from the UK National Registry of Childhood [91]. Most small GISTs are cured with surgery. However, TKI therapy improves the survival rate for patients with localised GIST as well as patients with advanced disease. The following three TKIs have been approved for the therapy of advanced GIST: imatinib, sunitinib, and regorafenib [92,93]. In the study of Agaram et al., 7 paediatric patients were treated with imatinib for 3 to 18 months (median: 5 months). In one patient, a stable disease response was observed. One patient had a mixed response in some nodules and slow progression in others. There were no therapeutic effects in the remaining four patients and sunitinib therapy was started. However, due to intolerance or progression of the disease, only one patient remained on sunitinib for 8 months and showed a stable disease response. Moreover, the small number of patients, the diversity of disease stages, sex, and age, heterogeneous entities, and the presence of genetic mutations did not allow for clear conclusions about the effectiveness of the TKI therapy [39]. Janeway et al. studied the use of sunitinib treatment in children with GIST who had failed prior imatinib therapy. Seven patients aged 10–17 years received sunitinib daily for four weeks. In six out of seven patients, stable disease or partial responses were observed. In five patients, the stabilisation time was longer with imatinib [51]. TKIs may have clinical efficacy in GIST, but large-group randomised trials are needed to establish treatment strategies.

### 6.2. Central Nervous System (CNS) Tumours

Embryonal carcinomas and germinomas are CNS germ cell tumours (GCT) and represent 3% of paediatric brain tumours in North America. Molecular studies have suggested that GCT may overexpress the proto-oncogene c-KIT [94]. In previous studies, TKIs have been used in GIST therapy where the c-KIT gene mutation was also present. Osorio et al. conducted a study in which dasatinib was used in the treatment of CNS germinoma. Due to the retrospective nature of the study, there are no unified data about the treatment effect, but four out of six patients remained disease-free for around 29–36 months after dasatinib therapy [95]. Another drug, gefitinib, was the first marketed inhibitor of EGFR. Due to the fact that EGFR expression has been noted in neuroblastoma, it was chosen for experimental treatment of CNS tumours. The results from three clinical trials of gefitinib in children’s CNS tumours suggested that gefitinib is safe and well-tolerated in a paediatric population [96,97,98]. The activity of gefitinib against CNS tumours has opened a new option for therapy for the future, but we are still waiting for data that show which treatment is the most advantageous [99].

### 6.3. Neuroblastoma

Neoplasms such as anaplastic large-cell lymphoma, inflammatory myofibroblastic tumours, non-small-cell lung cancer (NSCLC), and neuroblastoma have rearrangements within the oncogenic ALK gene. Based on this knowledge, it was hypothesised that the ALK inhibitor crizotinib may be a useful therapeutic strategy for their treatment [100,101,102]. Particular attention has been focused on neuroblastoma, which accounts for approximately 10% of all paediatric cancers and also accounts for up to 15% of deaths in children’s oncology [103,104,105,106]. In the study of Mossé et al., 79 children (median age: 10.1) with solid tumours or anaplastic large-cell lymphoma were treated with crizotinib. Of the 34 neuroblastoma patients, 11 had ALK mutations; in this group, one patient had a complete response and two had a stable disease response. In the other 23 patients with unknown ALK mutation status, 1 patient had a complete response, 5 patients had prolonged stable disease, and the remain on treatment. Although the preclinical data suggested that ALK mutations in neuroblastoma cells could be sensitive to ALK inhibition, additional studies with crizotinib are required to present clear evidence [107].

## 7. Paediatric Molecular Analysis for Therapeutic Choice (Paediatric MATCH)

**Figure 3:**
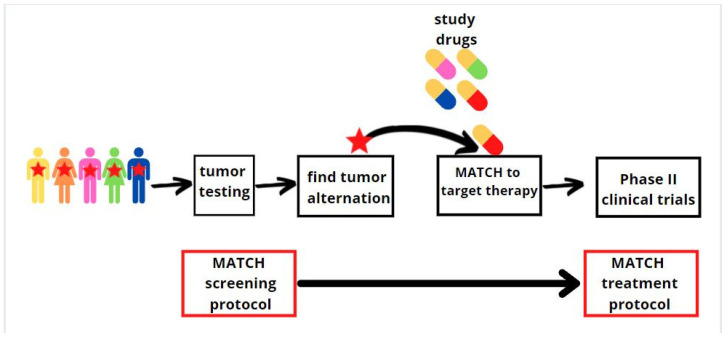
Graphics showing the MATCH protocol. Firstly, the patient is screened for mutations, and then the drug is selected for a specific mutation.

The field of precision medicine has exploded through progress in sequencing technologies. Identifying genetic abnormalities is critical to achieving better outcomes in paediatric malignancies, but the development of drugs that block specific proteins and pathways remains challenging. Paediatric cancers are rare and identifying a large group with specific genomic changes on which a clinical trial can be performed is a challenge [108]. Recurrent and refractory malignancies are rarely curable, which has forced the Children’s Oncology Group (COG) in partnership with the National Cancer Institute (NCI) to conduct a trial entitled the COG–NCI Paediatric Molecular Analysis for Therapeutic Choice (Paediatric MATCH) [109]. Paediatric MATCH is a unique histological trial where the treatment choice is based on a list of genomic aberrations that are known to be driver mutations for tumours and can be treated by the drug under investigation. This approach may be the basis for a better understanding of the pathogenesis and target points of targeted therapies for relapsed or refractory paediatric cancers. Firstly, in the NCI-MATCH study, the tumour tissues are analysed by the same analytically validated next-generation sequencing targeted assay for about 140 genes [110]. Genetic sequencing tests analyse the genetic material of the patients’ cancerous cells. Secondly, patients with genetic mutation changes receive an experimental treatment that is targeted at a specific genetic mutation of the tumour. Treatment continues for as long as the patient’s tumours are stable or are becoming smaller (Figure 3). Scientists plan to screen about 300 patients each year. Current research suggests that 10% of patients who have been screened will have a genetic change that matches one of the tested drugs. In the beginning, the study opened with seven treatment protocols; over the following year, three more protocols were added for a total of ten. Depending on the arm of the study, 13 different drugs have been used (Table 2). In the Parson study, a total of 422 patients aged 1 to 21 years old with refractory or solid tumours (71% of all cases), central nervous tumours (24%), or histiocytic disorders/lymphomas (5%) were sequenced for mutations. Actionable mutations were detected in 112 patients, and they were assigned to a treatment based on the Paediatric MATCH protocol [111]. Objective responses to treatment and patient outcomes are not known yet; thus, the clinical trial is still ongoing. Nevertheless, therapy that targets the gene mutations of the patients’ tumour holds great promise for revolutionising oncology.

## 8. Future Use of New TKIs in Cancer Treatment

Before the era of tyrosine kinase inhibitors, the treatment of malignancies in paediatric patients often failed, and outcomes were frequently poor. TKIs allowed patients to achieve greater overall survival and they can reduce or even eliminate many of the current chemotherapy problems, such as toxicity and drug resistance. International multicentre collaboration has increased the hope for new strategies, such as second- and third-generation TKIs, bi-specific monoclonal antibodies, and BCL-2 inhibitors [147]. One of the novel molecules is asciminib, an orally bioavailable first-in-class STAMP (specifically targeting the myristoyl pocket) *BCR-ABL1* inhibitor. Asciminib targets both native and mutated *BCR/ABL1*. As previously mentioned, TKIs’ mechanism of action focused on the ATP-binding site of kinase. However, asciminib is an allosteric inhibitor that binds a myristoyl site of the *BCR/ABL1* protein [148]. This other mechanism of action can be used in patients who are resistant to TKI due to ABL1-kinase domain mutations: *E255V G250E*, *Y253H*, *H396R*, and lastly, *T315I* [149]. Rea et al. have analysed CP-CML patients who had resistance or unacceptable side effects from at least two previous rounds of TKI therapy. The results were spectacular and showed that 92% (n = 34) of patients without a *T315I* mutation achieved CHR with asciminib [150]. Recently, research has increasingly used the synergy of TKI with chemotherapy or other PTKs such as MEK inhibitors. Such a combination can lower the toxicity of the therapy and improve the patient’s outcome. Zabkiewicz et al.’s study tested the ability of several TKIs, including pacritinib, to kill primary AML cells in a system that modelled primary AML and residual disease, providing clinically relevant therapeutic insights. Both pacritinib and ponatinib showed significantly greater sensitivity than crenolanib on primary AML blasts. Moreover, pacritinib did not produce any significant toxic effects on the stroma [151]. It seems quite likely that in the not-so-distant future, paediatric malignancies will be managed via a chemotherapy-free targeted treatment approach [152]. Research continues to shed light on the potential role of a STAMP inhibitor, and there are currently clinical trials using asciminib in Ph+ ALL and other *BCRL/ABL1* fusion-dependent malignancies [153].

## 9. Conclusions

Over the last years, remarkable advances in molecular science have helped us to understand the epigenetics of paediatric cancers and have highlighted major differences between paediatric and adult malignancies. Twenty years have passed since the approval of the first TKI, which started the target therapy era. The increasing numbers of TKIs have made treatment options based on patients’ individual genetic changes available and enlarged their chances for therapeutic success. Currently, TKIs are resulting in antitumour responses in historically difficult-to-treat malignancies, such as Ph+ ALL, CML, GIST, and CNS tumours. However, it should be remembered that satisfactory results of haematology therapy in adults are not always effective in children, and younger age is associated with more side effects. Paediatric patients, due to the genetic diversity and small size of groups with the same malignancies, are a challenge for conducting clinical trials, which are crucial for new therapeutic approaches. Future studies should focus on identifying molecular targets for precision therapy to establish effective management in paediatric oncology.

## Figures and Tables

**Table 1:** Tyrosine kinase inhibitors, their targets, and application in specific malignancies.

Generation of TKIs ^1^	Name	Chemical Structure	First FDA ^2^ Approval	Target	Disease	Children	Adults
I	Imatinib	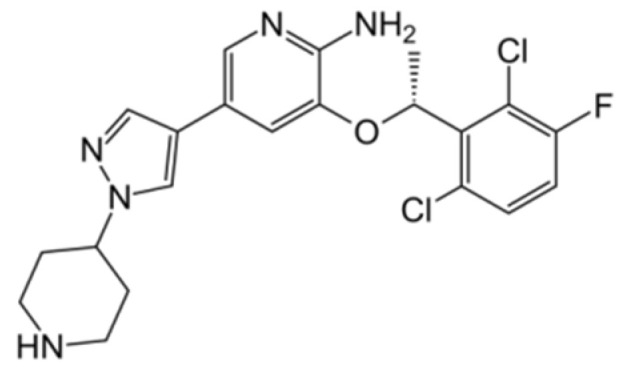	2001	BCR-ABL1 ^3^	CML ^4^	[33,34]	[35,36]
					Ph+ ALL ^5^	[37]	[38]
					GIST ^6^	[39]	[40,41]
					Brainstem gliomas, intracranial gliomas	[42]	[43]
	Crizotinib	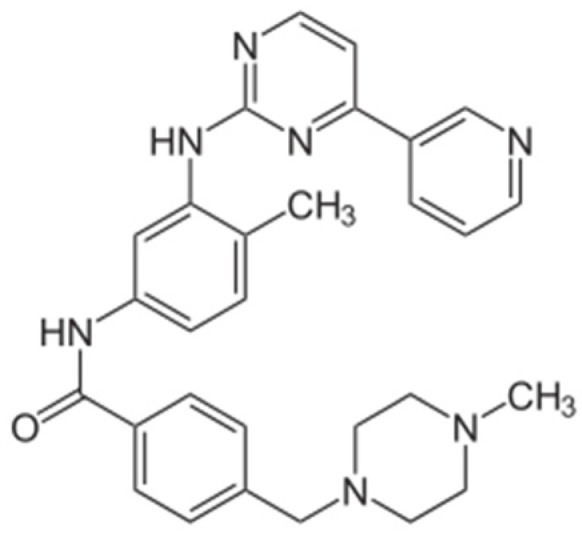	2011	ALK ^7^, ROS1 ^8^, c-MET ^9^	ALK-positive lung cancer	No data	[44]
					Intrinsic pontine glioma	[45]	No data
					Solid tumours (hepatocellular carcinoma, adrenal cortical carcinoma, Wilms’ tumour, neuroblastoma ganglioneuroblastoma, rhabdomyosarcoma, synovial sarcoma, epithelial myoepithelial carcinoma, Ewing sarcoma), anaplastic large-cell lymphoma	[46]	[47]
					Non-small-cell lung carcinoma	No data	[48]
	Sunitinib	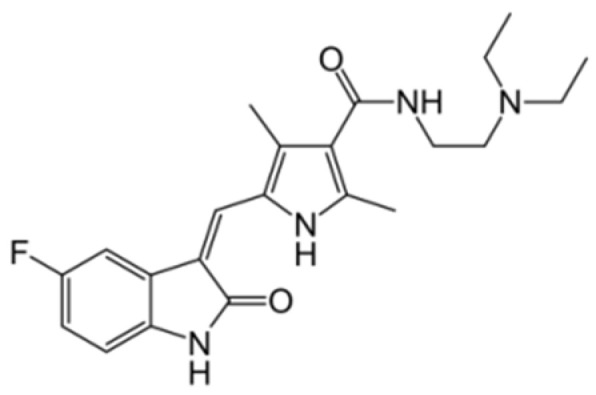	2006	VEGFR ^10^, VEGFR2 ^11^, VEGFR3 ^12^, c- KIT ^13^, FLT3 ^14^, CSF-1R ^15^, RET ^16^	Renal cell carcinoma	[49]	[50]
					GIST	[51]	No data
II	Regorafenib	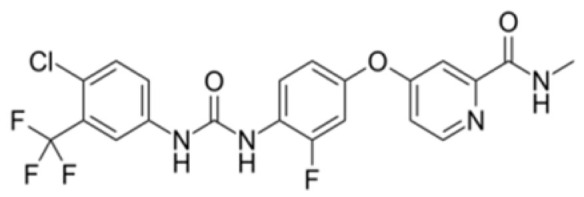	2012	VEGF, EGFR ^17^	Liposarcoma	Clinical trial number NCT02085148	[52]
					GIST		[53]
					Hepatocellular carcinoma		[54]
	Dasatinib	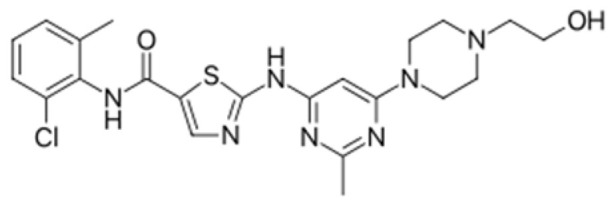	2006	BCR-ABL1, Src ^18^, c-Kit, ephrin receptors	CML	[55,56]	[57]
					Ph+ ALL	[58]	[57]
	Bosutinib	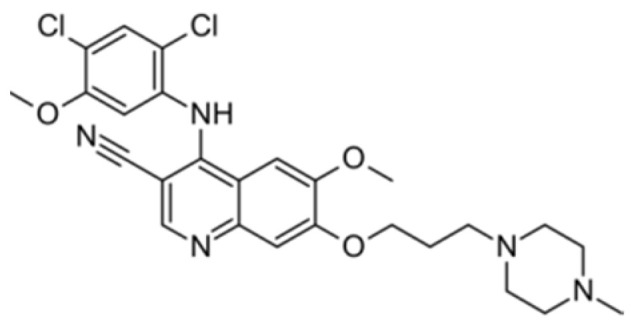	2012	BCR-ABL1, Src	CML	Clinical trial number NCT04258943	[41,59]
					Glioblastoma	No data	[60]
	Nilotinib	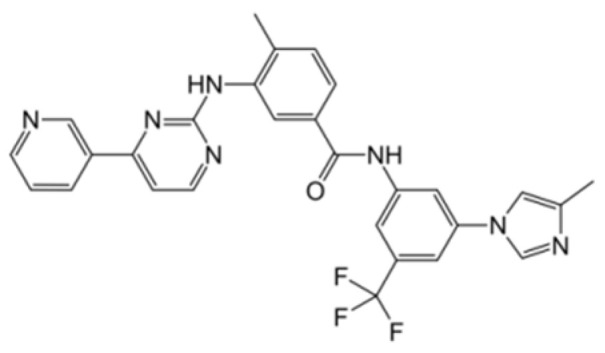	2007	BCR-ABL1	Ph+ ALL	No data	[58]
					CML	[61]	[62]
					GIST	No data	[63]
					Glioma	[64]	No data
III	Ponatinib	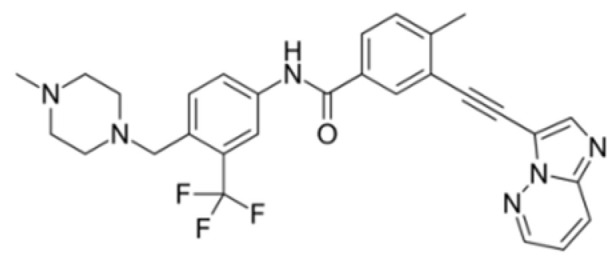	2012	BCR-ABL1	Glioblastoma	No data	[65]
					ALL ^19^	Clinical trial number NCT04501614	[66]
					AML ^20^	No data	[67]
					CML	[68]	[69]
	Osimertinib	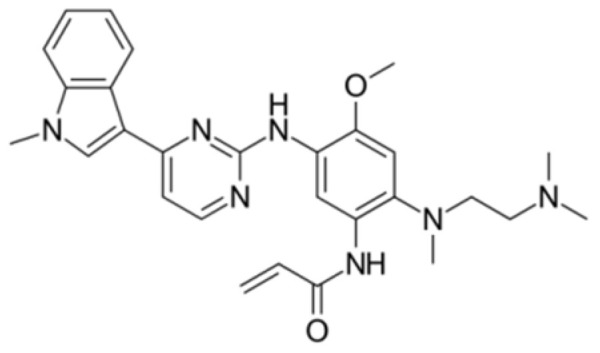	2020	EGFR	Non-small-cell lung carcinoma	No data	[70]
	Olmutinib	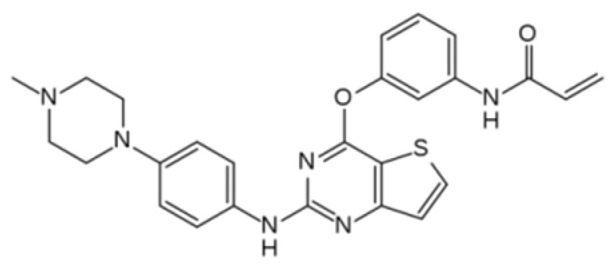	No FDA approval, approval in South Korea			No data	[71]
	Nazartinib	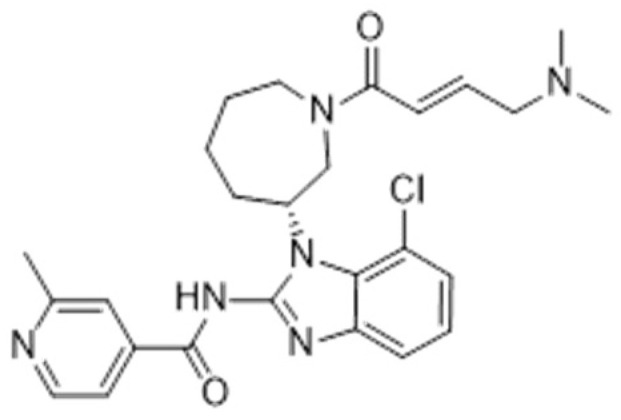	No approval, clinical trial number: NCT03040973		Non-small-cell lung carcinoma, advanced solid tumours	No data	[72]

^1^ TKI, tyrosine kinase inhibitor; ^2^ FDA, Food and Drug Administration; ^3^ BCR-ABL1, breakpoint cluster region and V-abl Abelson murine leukaemia viral oncogene homolog 1; ^4^ CML, chronic myeloid leukaemia; ^5^ Ph+ ALL, Philadelphia-chromosome-positive acute lymphoblastic leukaemia; ^6^ GIST, gastrointestinal stromal tumour; ^7^ ALK, anaplastic lymphoma kinase; ^8^ ROS1, ROS Proto-Oncogene 1, tyrosine kinase receptor; ^9^ c-MET, hepatocyte growth factor receptor; ^10^ VEGFR, vascular endothelial growth factor, ^11^ VEGFR2, vascular endothelial growth factor 2; ^12^ VEGFR3, vascular endothelial growth factor 3; ^13^ c-KIT, tyrosine protein kinase KIT; ^14^ FLT3, cluster of differentiation antigen 135; ^15^ CSF-1R, colony stimulating factor 1 receptor; ^16^ RET, proto-oncogene, rearranged during transfection; ^17^ EGFR, epidermal growth factor receptor; ^18^ Src, proto-oncogene, non-receptor tyrosine kinase; ^19^ ALL, acute lymphoblastic leukaemia; ^20^ AML, acute myeloid leukaemia.

**Table 2:** Mechanism of action and drugs in Paediatric MATCH.

Target	Drug	Used in the Treatment	References
NTRK1 ^1^, NTRK2 ^2^, or NTRK3 ^3^ gene fusion	Larotrectinib	Solid tumours, CNS ^4^ tumours, lung cancer, sarcomas, papillary thyroid cancer	[112,113,114,115]
FGFR1 ^5^, FGFR2 ^6^, FGFR3 ^7^, FGFR4 ^8^	Erdafitinib	Urothelial carcinoma, glioblastoma, endometrial cancer	[116,117,118]
Rb ^9^-positive, alterations in cell cycle genes	Palbociclib	Breast cancer, lung cancer, head and neck cancer, glioblastoma	[119,120,121,122,123]
ATM ^10^, BRCA1 ^11^, BRCA2 ^12^, RAD51C ^13^, RAD51D ^14^	Olaparib	Prostatic cancer, ovarian cancer, breast cancer	[124,125,126,127]
ALK ^15^ or ROS1 ^16^	Ensartinib	Non-small-cell lung carcinoma	[128,129]
TSC1 ^17^, TSC2 ^18^, PI3K/mTOR ^19^	Samotolisib	Preclinical studies only	[130]
MAPK ^20^ pathway mutations	Ulixertinib	Melanoma, colorectal cancer, lung cancer, non-small-cell lung carcinoma	[131,132]
BRAF ^21^ V600	Vemurafenib	Melanoma	[133,134]
Activating MAPK ^20^ pathway	Selumetinib sulphate	Melanoma, solid tumours	[135,136,137]
EZH2 ^22^, SMARCB1 ^23^, SMARCA4 ^24^	Tazemetostat	Follicular lymphoma, CNS tumours, solid tumours, B-cell non-Hodgkin lymphoma	[138,139,140]
HRAS ^25^ gene alterations	Tipifarnib	Salivary gland cancer, breast cancer, AML^26^, myelodysplastic syndrome, solid tumours	[141,142,143,144]
Activating RET ^27^ mutations	Selpercatinib	Non-small-cell lung cancers, thyroid cancer	[145,146]

^1^ NTRK1, non-receptor protein tyrosine kinase 1; ^2^ NTRK2, non-receptor protein tyrosine kinase 2; ^3^ NTRK3, non-receptor protein tyrosine kinase 3; ^4^ CNS, central nervous system; ^5^ FGFR1, fibroblast growth factor receptor 1; ^6^ FGFR2, fibroblast growth factor receptor 2; ^7^ FGFR3, fibroblast growth factor receptor 3; ^8^ FGFR4, fibroblast growth factor receptor 4; ^9^ Rb retinoblastoma; ^10^ ATM, ATM serine/threonine kinase; ^11^ BRCA1, breast cancer 1 gene; ^12^ BRCS2, breast cancer 2 gene; ^13^ RAD51C, RAD51 *S. cerevisiae* homolog C; ^14^ RAD51D, RAD51 homolog D (*S. cerevisiae*); ^15^ ALK, anaplastic lymphoma kinase; ^16^ ROS1 ROS proto-oncogene 1; ^17^ TSC1, tuberous sclerosis 1; ^18^ TSC2, tuberous sclerosis 2; ^19^ PI3K/mTOR, phosphatidylinositol 3-kinase/mammalian target of rapamycin kinase; ^20^ MAPK, mitogen-activated protein kinase; ^21^ BRAF, gene encodes protein B-Raf; ^22^ EZH2, enhancer of zeste homolog 2; ^23^ SMARCB1, SWI/SNF-related, matrix-associated, actin-dependent regulator of chromatin, Subfamily B, Member 1; ^24^ SMARCA4, SWI/SNF-related, matrix-associated, actin-dependent regulator of chromatin, Subfamily A, Member 4; ^25^ HRAS v, Ha-ras Harvey rat sarcoma viral oncogene homolog; ^26^ AML, acute myeloid leukaemia; ^27^ RET, “rearranged during transfection” proto-oncogene.

## Data Availability

No new data were created or analysed in this study. Data sharing is not applicable to this article.

## Abbreviations

ABL	V-abl Abelson murine leukaemia viral oncogene homolog 1
Akt	serine/threonine kinase 1
ALK	anaplastic lymphoma kinase
ALL	acute lymphoblastic leukaemia
AML	acute myeloid leukaemia
ATM	ATM serine/threonine kinase
ATP	adenosine triphosphate
BCR	ABL1-breakpoint cluster region and V-abl Abelson murine leukaemia viral oncogene homolog
BRAF	gene encoding protein B-Raf
BRCA1	breast cancer 1 gene
BRCA2	breast cancer 2 gene
CCyR	complete cytogenetic response
CDK4/6	cyclin-dependent kinase
CHR	complete haematologic response
c-KIT	tyrosine-protein kinase KIT
c-MET	hepatocyte growth factor receptor
CML-BP	chronic myeloid leukaemia plastic phase
CML	chronic myeloid leukaemia
CNS	central nervous system
COG	Children’s Oncology Group
CSF-1R	Colony stimulating factor 1 receptor
EGFR	epidermal growth factor receptor
ELN	European Leukaemia Net
ERK1/2	extracellular signal-regulated kinases
EsPhALL	European Study of Postinduction Treatment of Ph-Positive ALL
EZH2	enhancer of zeste homolog 2
FDA	Food and Drug Administration
FGFR1	fibroblast growth factor receptor 1
FGFR2	fibroblast growth factor receptor 2
FGFR3	fibroblast growth factor receptor 3
FGFR4	fibroblast growth factor receptor 4
FLT3	cluster of differentiation antigen 135
GCT	germ cell tumour
GIST	gastrointestinal stromal tumour
HER2	human epidermal growth factor receptor 2
HRAS	v-Ha-ras Harvey rat sarcoma viral oncogene homolog
HSCT	haematopoietic stem cell transplantation
IRIS	International Randomised Study of Interferon and STI571
KIT	tyrosine-protein kinase KIT cluster of differentiation 117
MATCH	Molecular Analysis for Therapeutic Choice
MAPK	mitogen-activated protein kinase
MET	MET proto-oncogene, tyrosine kinase receptor
MM-GBMV	molecular mechanics—generalised Born molecular volume
MMR	major molecular response
NCI	National Cancer Institute
NCI-MATCH	National Cancer Institute—Molecular Analysis for Therapeutic Choice
NRTK	non-receptor protein tyrosine kinase
NSCLC	non-small-cell lung cancer
OS	overall survival
P loop	phosphate binding loop
P13K/mTOR	intracellular signalling pathway
pan-TRK	family of receptor tyrosine kinases
PARP	poly (ADP-ribose) polymerase
PDGFRα	platelet-derived growth factor receptor alpha
PDGFRβ	platelet-derived growth factor receptor beta
Ph-like ALL	Philadelphia chromosome-like acute lymphoblastic leukaemia
Ph+ ALL	Philadelphia chromosome acute lymphoblastic leukaemia
PI3K	phosphoinositide 3-kinase
PI3K/mTOR	phosphatidylinositol 3-kinase/mammalian target of rapamycin kinase
PTK	protein tyrosine kinase
Rb	retinoblastoma
RAF	proto-oncogene
RAS	rat sarcoma virus protein family
Rb	retinoblastoma
RET	“rearranged during transfection” proto-oncogene
RR	relative resistance
ROS1	ROS proto-oncogene 1, tyrosine kinase receptor
RTK	receptor protein tyrosine kinase
SMARCB1	SWI/SNF-related, matrix-associated, actin-dependent regulator of chromatin, Subfamily B, Member 1
SMARCA4	SWI/SNF-related, matrix-associated, actin-dependent regulator of chromatin, Subfamily A, Member 4
SRC	Src proto-oncogene, non-receptor tyrosine kinase
STAMP	specifically targeting the myristoyl pocket
STI571	imatinib
TKI	tyrosine kinase inhibitor
TRKA	tropomyosin receptor kinase A
TRKB	tropomyosin receptor kinase B
TRKC	tropomyosin receptor kinase C
TSC1	Tuberous sclerosis 1
TSC2	Tuberous sclerosis 2
UK	United Kingdom
USA	United States of America
VEGFR	vascular endothelial growth factor
VEGFR2	vascular endothelial growth factor 2
VEGFR3	vascular endothelial growth factor 3
